# TGF-β and microRNA Interplay in Genitourinary Cancers

**DOI:** 10.3390/cells8121619

**Published:** 2019-12-12

**Authors:** Joanna Boguslawska, Piotr Kryst, Slawomir Poletajew, Agnieszka Piekielko-Witkowska

**Affiliations:** 1 Department of Biochemistry and Molecular Biology, Centre of Postgraduate Medical Education; 01-813 Warsaw, Poland; joanna.boguslawska@cmkp.edu.pl; 2 II Department of Urology, Centre of Postgraduate Medical Education, 01-813 Warsaw, Poland; piotr.kryst@cmkp.edu.pl (P.K.); slawomir.poletajew@gmail.com (S.P.)

**Keywords:** genitourinary cancers, renal cancer, penile cancer, testicular cancer, bladder cancer, prostate cancer, TGF-β, microRNA, treatment, diagnosis

## Abstract

Genitourinary cancers (GCs) include a large group of different types of tumors localizing to the kidney, bladder, prostate, testis, and penis. Despite highly divergent molecular patterns, most GCs share commonly disturbed signaling pathways that involve the activity of TGF-β (transforming growth factor beta). TGF-β is a pleiotropic cytokine that regulates key cancer-related molecular and cellular processes, including proliferation, migration, invasion, apoptosis, and chemoresistance. The understanding of the mechanisms of TGF-β actions in cancer is hindered by the “TGF-β paradox” in which early stages of cancerogenic process are suppressed by TGF-β while advanced stages are stimulated by its activity. A growing body of evidence suggests that these paradoxical TGF-β actions could result from the interplay with microRNAs: Short, non-coding RNAs that regulate gene expression by binding to target transcripts and inducing mRNA degradation or inhibition of translation. Here, we discuss the current knowledge of TGF-β signaling in GCs. Importantly, TGF-β signaling and microRNA-mediated regulation of gene expression often act in complicated feedback circuits that involve other crucial regulators of cancer progression (e.g., androgen receptor). Furthermore, recently published in vitro and in vivo studies clearly indicate that the interplay between microRNAs and the TGF-β signaling pathway offers new potential treatment options for GC patients.

## 1. Introduction

Transforming growth factor-beta (TGF-β) emerges as one of the key regulators of tumor development and progression. It influences all crucial steps of cancer progression, including migration and invasion, with a prominent influence on the process of epithelial–mesenchymal transition (EMT). The mechanisms of TGF-β actions in cancer are complex. At the beginning of tumor development, TGF-β attenuates cancerous proliferation, limiting tumor growth. During cancer progression, these tumor suppressive effects reverse, and TGF-β starts to promote migration, invasion, and formation of distant metastasis [1]. This so called “TGF-β paradox”, universally occurring in cancers, has remained a mystery for many years. Recently, a growing body of evidence suggests that these paradoxical TGF-β actions could result from interplay with the activity of microRNAs, which emerge as important modulators and mediators of TGF-β effects in cancer cells. These small, non-coding RNAs are involved in the control of a wide array of critical biological processes, including cells development, proliferation, growth, differentiation, and apoptosis. Abnormalities in their expression or function contribute to the development of multiple disorders, including cancer [2]. microRNAs are transcribed as long, hairpin-shaped primary transcripts called pri-microRNAs. They are further processed and cleaved by Drosha ribonuclease and DGCR8 RNA binding protein, which form the microprocessor complex. The resulting pre-microRNA is exported outside the nucleus by exportin 5 for further processing in the cytoplasm where RNase dicer cleaves it into mature, short, double-stranded miRNA complex. Next, one of the miRNA strands is assembled with argonaute 2 (Ago2) protein within the RISC (RNA-induced silencing) complex, where miRNA functions as a guide enabling targeting mRNA to be degraded or translationally repressed by Ago2. The expression and functioning of miRNAs are modulated by multiple factors, including TGF-β, which regulates microprocessor activity by recruiting SMAD proteins, which enhance Drosha-mediated processing of pri-miRNA [3].

Genitourinary cancers (GCs) represent 25% of all solid tumors [4]. They derive from different types of cells located in the kidney, penis, testis, bladder, and prostate [5,6]. GCs are highly divergent, in terms of molecular pathology and prognosis, ranging from excellent outcomes for patients with testicular cancer to metastatic clear cell renal cell carcinoma, which is associated with poor outcomes. Recent studies revealed the importance of TGF-β signaling in GCs, including animal in vivo studies, bringing hope for new therapeutic approaches. On the other hand, multiple studies showed that microRNAs play an important role in the development and progression of many types of genitourinary tumors. Here, we discuss the interplay between TGF-β and microRNAs and demonstrate its significance as a new field for therapeutic interventions in genitourinary cancers.

## 2. Genitourinary Cancers (GC): Subtypes, Treatment, and Prognosis

Genitourinary cancers encompass a large group of tumors deriving from different types of cells in the kidney, bladder, prostate, testis, and penis. The particular GC types differ in molecular pathology and biological behavior, which leads to variant treatment options and prognosis. While in patients with testicular or bladder cancer there is a need for urgent aggressive multimodal treatment, in many cases of prostate or renal cancer, active surveillance can be a safe option. Although surgery plays a fundamental role in the radical treatment of all genitourinary cancers, it can be complemented by local or systemic chemotherapy, radiation therapy, local or systemic immunotherapy, hormonal therapy, or targeted therapies.

### 2.1. Renal Cancer

Renal tumors in adults arise mainly from the epithelium lining renal tubules and are classified as renal cell carcinomas (RCCs). From a histological perspective, there are three main types of RCCs: Clear cell RCC (ccRCC), papillary RCC (pRCC), and chromophobe RCC (chRCC). Novel RCC subtypes have recently been defined based on molecular features (e.g., succinate dehydrogenase (SDH)–deficient renal carcinomas) [5]. They differ in genetics and prognosis, with ccRCC being both the most common and the most aggressive one [7,8]. The key molecular alteration in ccRCC is von Hippel–Lindau (*VHL*) gene mutation, which leads to uncontrolled hypoxia-induced factor (HIF) expression, followed by the activation of several growth factor pathways, including vascular endothelial growth factor (VEGF), platelet-derived growth factor (PDGF), and others [9].

With increasing use of imaging studies, nowadays, the majority of renal masses are diagnosed incidentally at a low stage [10]. Treatment of choice consists of surgical removal of the tumor, namely partial or radical nephrectomy [11]. The surgery can be either curative or cytoreductive in selected metastatic cases [12,13]. In metastatic disease, patients can be treated with systemic targeted therapy and/or immunotherapy. Targeted therapies act mainly via the VEGF pathway. Tyrosine kinase inhibitors (TKIs) are the most commonly used group, including sunitinib, pazopanib, cabozantinib, and others [11]. Regarding immunotherapy, monotherapy or the combination of nivolumab and ipilimumab are effective treatment options [14]. Nivolumab, an anti-PD1 antibody, restores antitumor activity of T cells while ipilimumab, an anti-CTLA4 antibody, potentiates this activity [15,16].

Prognosis of patients with RCC has significantly improved in the last years due to the progress in systematic therapies, including targeted anti-VEGF therapies and immunotherapies. In general, the 5-year overall survival in renal cancer patients is 49% [17,18]. Cancer-specific survival (CSS) depends on the tumor histology and stage of the disease. For ccRCC, 5-year CSS is estimated as 71% while it is much higher for chRCC and pRCC, reaching 88% and 91%, respectively [19]. Depending on the clinical stage, 5-year CSS in ccRCC can drop from 91% in clinical stage I to 32% in clinical stage IV [20].

### 2.2. Penile Cancer

Penile cancer is a rare tumor, accounting for about 0.2% of all cancer cases [21]. Usually, it is a squamous cell carcinoma arising from the glans penis or the inner prepuce of foreskin [22]. One-third of cases are associated with the human papilloma virus (HPV) infection, especially by so-called high-risk HPV genotypes 16, 18, 33, and 35 [23].

Treatment of patients with penile cancer covers two aspects: Primary lesion and lymph node management. Treatment of primary lesion requires surgery, with organ-sparing procedures now a standard of care whenever feasible and oncologically safe [24,25]. There are also alternatives to surgery, including topical treatments or laser ablation in low-stage and low-grade cases or radiation therapy in cases with small lesions [26,27,28,29]. Lymph node status and management have the strongest impact on patients’ survival [22]. In patients with clinically enlarged inguinal +/− pelvic lymph nodes, lymph node dissection (LND) is clearly indicated. In patients with clinically negative lymph nodes, surveillance can be considered in low-risk cases while invasive nodal staging with limited LND or sentinel node biopsy should be performed in the remaining cases [22]. In advanced and metastatic cases, systemic cisplatin-based chemotherapy is the treatment of choice [30].

For prognosis, stage of the disease, tumor size, and ethnicity were recently reported to be the most significant factors [31]. In general, 5-year overall survival in penile cancer patients is 61% [32]. However, the cancer-specific death rate does not exceed 19%, even in patients aged <40 years [33].

### 2.3. Testicular Cancer

Testicular cancer (TC) is a relatively rare tumor, representing approximately 1% of male neoplasms in the general population [34]. However, in a subset of young men, the incidence of testicular cancers is increasing, with 3 to 10 new cases diagnosed per 100,000 males/per year in industrialized countries [34]. The vast majority (90%–95%) of TCs are classified as testicular germ cell tumors (TGCTs), which are the most common malignancies diagnosed in males aged 15 to 44 years [35,36]. TGCTs develop from cells of germ cell neoplasia in situ (GCNIS), which originate from gonocytes that fail to undergo physiological spermatogenic differentiation [35]. They are commonly further divided into seminomas and non-seminoma tumors based on their histology and radiosensitivity [5]. An isochromosome of the short arm of chromosome 12 (12p) is a specific genetic marker of all GCTs [37]. Among numerous mutations described in testicular GCTs, the *TP53* mutation is the most common [38].

The treatment of TC has been called one of the top five advances in 50 years of modern oncology [39]. Treatment usually starts with surgery, namely radical orchiectomy. In very selected cases, testis sparing surgery is an option to preserve hormonal and reproductive function of the gonad [40]. Excellent cure rates result mainly from the efficacy of systemic chemotherapy, usually based on cisplatin [41]. Testicular tumors are chemosensitive while seminomas are also radiosensitive. The vast majority of orchiectomized patients are candidates for adjuvant chemotherapy while final qualification is based on stage of the disease and estimated risk of relapse [42,43,44]. In clinical stage II seminomas, radiation therapy is an alternative to chemotherapy. Finally, retroperitoneal lymph node dissection can be an option for patients relapsing after chemotherapy or with residual retroperitoneal disease after chemotherapy or in the case of contraindications to chemotherapy [40].

Prognosis depends mainly on the stage of the disease, including the presence and location of metastases and the serum concentration of biomarkers after orchiectomy (alpha-fetoprotein (AFP), human chorionic gonadotropin (hCG), lactate dehydrogenase (LDH)). Depending on these factors, the 5-year overall survival in patients with metastatic disease ranges from 92% in the good prognosis group to 48% in the poor prognosis group of non-seminoma patients [44]. At the same time, the vast majority of testicular cancer cases are non-metastatic patients with a very good prognosis [45].

### 2.4. Bladder Cancer

Bladder cancer (BC) is the most common malignancy within the urinary tract [21]. The annual BC incidence reaches nearly 10 cases per 100,000 persons in developed regions, with 430,000 diagnosed cases and nearly 170,000 deaths annually worldwide [46]. The vast majority (up to 75%) of these tumors are urothelial carcinomas, arising from urothelium in the process of multistep heterogeneous mutations [6,47,48,49]. From a biological and clinical standpoint, BC is classified into non-muscle invasive (NMIBC), representing 70% to 80% of BC cases, and muscle invasive bladder cancer (MIBC) [50]. These two entities differ in terms of incidence, gene mutations, morphology, and aggressiveness [51,52,53,54]. Cases of NMIBC are further divided into three risk groups (low, intermediate, high) depending on the risk of recurrence and progression after resection [55,56]. While NMIBCs can be radically treated by endoscopic resection with or without adjuvant intravesical chemo- or immunotherapy, MIBCs require major surgery, namely radical cystectomy (removal of the urinary bladder, prostate, seminal vesicles, and pelvic lymph nodes in men; removal of the urinary bladder, uterus, adnexa, anterior wall of the vagina, and pelvic lymph nodes in women) with perioperative chemotherapy [50].

Up to 15% of bladder cancer patients are diagnosed upfront with metastatic disease [57,58]. In these cases, surgery is no longer a standard option and systemic, preferentially cisplatin-based, chemotherapy is the treatment of choice [59]. As neoantigen load and T cell infiltration in bladder cancers is high [60], new systemic treatment options with check-point inhibitors were shown to be effective [61]. Until now, the European Medical Agency has registered pembrolizumab, atezolizumab, and nivolumab for treatment of patients with advanced bladder cancer. Many further phase II and phase III trials are ongoing.

Prognosis in bladder cancer depends mainly on the stage of the disease. The survival rate in NMIBC is high while the 5-year risk of recurrence and progression after endoscopic resection reaches 31% to 78% and 1% to 45%, respectively [55]. Bladder sparing is possible and safe in the majority of these patients; however, they all require a close follow-up with repeated cystoscopies to detect disease relapse early [50]. On the contrary, prognosis in MIBC is poor. The 5-year recurrence rate after radical cystectomy in MIBC patients is 32% to 68% [62,63,64]. Systemic first-line chemotherapy in advanced and metastatic MIBCs offers a response rate of 46% to 49% and median overall survival of 14 to 15 months [65]. Unfortunately, over 50% of MIBC patients are cisplatin ineligible due to reduced renal function, performance status, heart failure, or other comorbidities [66]. Patients unfit for cisplatin or progressing after cisplatin therapy can be candidates for second-line treatment modalities, including carboplatin-based regimens, check-point inhibitors, monotherapy, or best supportive care.

### 2.5. Prostate Cancer

Prostate cancer (PCa) is the most common malignancy among men in America and Europe [21]. It is an androgen-dependent cancer, which develops typically in the peripheral zone of the prostate [67]. The introduction of PSA testing has dramatically increased diagnosis, with a limited positive impact on patients’ survival in the long run [68,69,70]. Prostate cancer is a disease of a different genetic mutation spectrum, different histological tumor grades, different progression potential, and different treatment options.

Standard curative treatment of prostate cancer patients involves surgery (radical prostatectomy) or radiation therapy (external beam radiation or/and brachytherapy) [71]. However, in many low- volume and low-grade cancers, potential progression is so slow that observation can be a safe alternative [72,73]. This is called active surveillance if radical treatment is considered or watchful waiting if only palliative therapy is an option [43,74,75]. In advanced cases, when radical treatment cannot be implemented, androgen deprivation therapy (ADT) is the treatment of choice. It involves bilateral orchiectomy or pharmacological castration with androgen receptor antagonists, GnRH agonists, or GnRH antagonists. In upfront metastatic patients, ADT should be combined with docetaxel chemotherapy or abiraterone [76,77,78,79,80]. At later disease stages, when prostate cancer is no longer castration sensitive (castration-resistant prostate cancer), there are several second-line treatment options, including chemotherapy (docetaxel and cabazitaxel), new antiandrogens (enzalutamide, apalutamide), steroidogenesis inhibitor (abiraterone), immunotherapy (sipuleucel-T), or radioactive compounds (radium 223) [81].

Prognosis in prostate cancer patients depends mainly on the stage of the disease and tumor grade. As these patients are usually elderly men with comorbidities, many of them die with prostate cancer but not from the cancer. Outcomes of local treatment in organ-confined disease by surgery and radiation therapy are described by a 5-year biochemical recurrence rate of 27% to 53% [81]. However, subsequent salvage therapy leads to undetectable PSA levels in the majority of patients. Moreover, many of the men with biochemical recurrence will never experience clinical progression [81]. In patients with advanced cancer, median overall survival depends on the response to ADT as measured by a PSA level decrease. In patients with a good response (PSA level after 7 months of ADT <0.2 ng/mL), it is 75 months while in patients with a poor response (PSA > 4.0 ng/mL), it is 13 months [82].

## 3. The Basics of TGF-β Signaling

**Figure 1:**
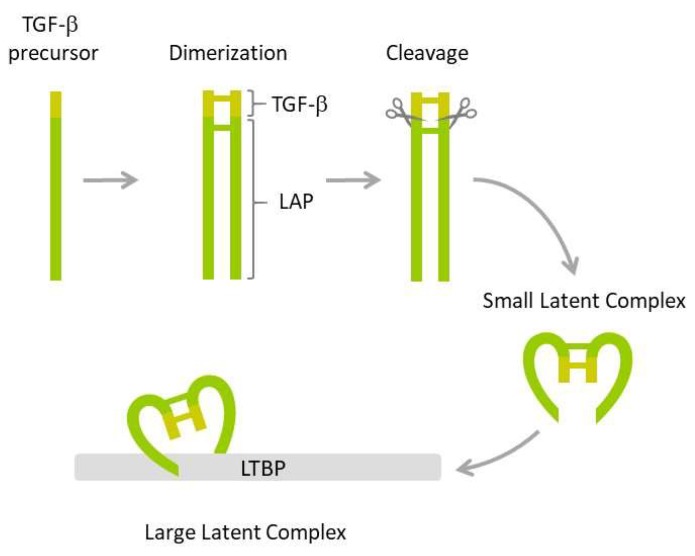
TGF-β maturation. The inactive TGF-β precursor dimerizes and the resulting dimer is cleaved by furin endopeptidase resulting in mature TGF-β and the latency-associated peptide (LAP) that bind non-covalently to produce small latent complex. The latter is next bound by latent TGF-β binding protein (LTBP), resulting in a large latent complex.

The family of transforming factor β (TGF-β) proteins is a large group of 33 structurally related growth factors, including notably TGF-β1, TGF-β2, TGF-β3, activins/inhibins, bone morphogenetic proteins (BMPs), and growth differentiation factors (GDFs). TGF-β1 is the most studied isoform expressed in mammalian tissues. TGF-β1 is expressed as an inactive precursor protein that undergoes a series of posttranslational modifications (Figure 1).

The first step involves dimerization of two precursors and subsequent cleavage by furin endopeptidase, which produces two smaller proteins: The latency-associated peptide (LAP) and the mature TGF-β1. The two proteins form a non-covalently bound small latent complex in which TGF-β1 is enclosed and protected by the surrounding LAP. Finally, the small latent complex is covalently bound by latent TGF-β binding protein (LTBP) to form a large latent complex, which is secreted by the cell. Both LAP and the formation of latent complexes are required for proper folding, maturation, and secretion of TGF-β1. They also ensure TGF-β stability and prevent its inappropriate activation. TGF-β cytokine is activated by several mechanisms, including those triggered by plasmin, thrombospondin-1, integrins, matrix metalloproteinases, calpains, retinoic acids, fibroblast growth factor-2, and reactive oxygen species (ROS) [83,84].

TGF-β initiates intracellular signaling by binding to its cell surface receptor, TGFBR2, which forms a heterotetrameric complex with TGFBR1. This results in serine/threonine phosphorylation of the cytoplasmic GS domain of TGFBR1 and initiation of signaling cascades. In the canonical mechanism of TGF-β action, activation of TGFBR1 leads to phosphorylation of SMAD2/3 proteins that interact with SMAD4 and, following translocation to the nucleus, regulate transcription of TGF-β-target genes. Recruitment of SMAD2/3 to the TGF-β receptors is mediated by SARA protein. TGF-β signaling is negatively regulated by SMAD6 and SMAD7, which interfere with the activation of receptors, formation of the SMAD2/3-SMAD4 complex, and inhibit transcription of TGF-β-target genes by binding to the DNA sequence in their promoter regions [85].

In the non-canonical pathway, TGF-β activates ubiquitin ligase TRAF6 (tumor necrosis factor receptor-associated factor 6) that ubiquitinates TGFBR1, which in turn results in selective proteolytic cleavage by TACE and PS1 (presenilin), leading to liberation of the intracellular domain of TGFBR1. The relieved domain is translocated to the nucleus where it interacts with various molecules and activates multiple signaling pathways, including these involving Ras, RHOA (Ras homolog family member A), PI3K (phosphoinositide 3-kinase), PP2A (protein phosphatase 2A), MAPK (mitogen-activated protein kinase), TAKI1 (TGF-β-activated kinase), ERK1/2 (extracellular signal-regulated kinase ½), and JNK (c-Jun N-terminal kinase) [86,87]. The described mechanisms of TGF-β action are also regulated by multiple accessory receptor proteins, transcriptions factors, and transcriptional co-factors in a cell type- or context-specific manner. TGF-β-induced signaling contributes to the regulation of cell growth and differentiation, apoptosis, cell motility, production of the extracellular matrix, angiogenesis, and immunity.

TGF-β plays a dual role in carcinogenesis. In the early stages of tumor development, it inhibits cellular transformation and prevents cancer progression while in later stages, TGF-β plays the oncogenic role and promotes tumor progression by induction of epithelial–mesenchymal transition (EMT), stimulation of angiogenesis, and immunosuppression. This conversion in cancer-related TGF-β functions is known as the “TGF-β paradox” and has been described in detail in previous publications [1,86,87,88,89,90,91,92]. These dichotomous TGF-β actions occur universally in tumors, including breast [93], liver and gastrointestinal [94], colorectal [95], pancreatic [96], or lung cancers [97]. It appears that as tumors progress, cancer cells tend to acquire resistance to TGF-β growth inhibitory effects due to mutations and/or functional inactivation of TGF-β pathway elements. The most commonly mutated genes of the TGF-β signaling pathway include *TGFBR1*, *TGFBR2*, *SMAD4*, and *SMAD2*. The significance of the TGF-β signaling pathway in cancer was recently confirmed by a report of TCGA (The Cancer Genome Atlas) consortium. The analysis of tissue samples from 33 cancer types and > 9000 patients revealed that the genes encoding the TGF-β pathway elements were impaired in nearly 40% of the analyzed cancer cases [98]. Remarkably, in a subset of tumors that included genitourinary cancers (bladder urothelial carcinoma, clear cell renal cell carcinoma, papillary renal cell carcinoma, prostate adenocarcinoma, and testicular germ cell tumor), the activity of the TGF-β signaling pathway correlated with epithelial–mesenchymal transition. The same study also showed that miRNAs contribute to the transcriptional activity of the TGF-β pathway, indicating functional links between short-non-coding RNAs and transforming growth factor effects in cancer cells.

## 4. Alterations in TGF-β Signaling in Genitourinary Cancers

### 4.1. Renal Cancer

The proteins of the TGF-β family play an important role during kidney development and functioning. The gene encoding TGF-β is expressed in mammalian metanephros during tubular development [99]. Activin A and TGFB2 are involved in the formation of the ureteric bud [100,101] while TGFB2 knock-out results in kidney agenesis in mice [102]. 

TGF-β is a critical mediator of renal fibrosis by promoting intense production and accumulation of components of the extracellular matrix (ECM), resulting in renal damage [103]. Renal fibrosis contributes to development of chronic kidney diseases (CKDs), which in turn may progress to end-stage renal diseases (ESRDs). The role of TGF-β in renal fibrosis and other chronic kidney diseases was extensively reviewed [104,105,106,107]. Remarkably, despite the well-documented role of TGF-β1 in renal fibrosis, the role of TGF-β in renal cancer remains elusive due to the conflicting results of published studies.

RCC-derived cell lines and tumors consistently express and secrete increased amounts of TGF-β1 [108,109,110,111,112,113] (Table 1). 

Increased TGF-β1 levels were also found in plasma and serum [114,115,116], as well as peripheral blood and tumor infiltrating lymphocytes (TILs) [117] of RCC patients. TGF-β1 expression directly correlates with tumor stage and grade, and is significantly increased in patients with metastatic RCC [116], indicating its importance in tumor progression [137]. Contradictory results were presented by Zheng et al., who demonstrated downregulation of TGF-β1 expression in metastatic renal cancer tissue compared to primary tumor samples, suggesting DNA hypermethylation as a possible cause of decreased expression. These results were recapitulated by a metastatic and primary renal cancer mice xenograft model. Pretreatment of cancer cells with 5-aza-2’-deoxycytidine (DNA methylation inhibitor) before inoculation in mice led to increased TGF-β1 expression, tumor size reduction, and an extended survival time of animals [138].

Sjolund et al. proved that elevated levels of TGFBR1 correlated with worse prognosis for ccRCC patients [139]. Similar results were obtained by Sitaram et al., who analyzed the expression of TGFBR1-FL (the full-length receptor) and TGFRB1-ICD (the intracellular domain of TGFRB1 receptor) in RCC [140]. Moreover, an analysis of 151 cases of urinary system cancers provided evidence that an intronic variant of TGFBR1, Int7G24A (rs334354), was associated with a higher risk of RCC development [141]. TGFBR2 expression was shown to decrease with cancer progression [142,143], which can partly explain the resistance of RCC cells to the TGF-β1 growth-inhibiting effect. However, in earlier research, conflicting findings showed that downregulation of *TGFBR2* expression is associated with a better prognosis for ccRCC patients [144]. Similar results were obtained by Kominsky et al., who observed that reducing *TGFBR2* expression and blocking the TGF-β pathway inhibited ccRCC metastasis to bone [145]. Furthermore, Ananth et al. demonstrated inactivation of TGF-β signaling in a 786-0 ccRCC-derived cell line due to the loss of the TGRBR2 receptor, whereas Sjolund et al. provided evidence that this pathway is functional in the same cell line [139,146]. Another study demonstrated that TGFBR3 has anticancer properties in ccRCC independent of TGF-β and its canonical mechanism of action, and that loss of this receptor may occur early during RCC carcinogenesis [143]. In accordance, downregulation of TGFBR3 expression in primary ccRCC was associated with poor survival of patients [139]. Recently, downregulation of TGFBR3 expression in advanced ccRCC tumor samples was confirmed by an independent study in which the loss of this receptor led to the stimulation of cell migration and formation of lung metastasis [147].

The expression and role of other components of the TGF-β signaling pathway in RCC were also investigated. Immunohistochemical analysis of 637 ccRCC tissue samples revealed a negative correlation between expression of SMAD3 and SMAD4, and the age of patients, nuclear grade, tumor size, as well as pTNM stage. Moreover, the expressions of these proteins were independent indicators of patients’ progression-free survival [148]. Increased expression of pSMAD2/3 (phosphorylated SMAD2 and SMAD3) and PAI-1 (plasminogen activator, TGF-β1 target gene) was observed in more differentiated ccRCC tumor samples and correlated with a larger tumor size and lower patient survival rate. Furthermore, stimulation of ccRCC cell lines, 786-0 and A498, with the exogenous TGF-β1 triggered activation of the TGFBR1 and TGF-β/SMAD/PAI-1 pathway and stimulated the invasive potential of ccRCC cells [140].

*VHL* inactivation is the key molecular aberration associated with ccRCC and several studies demonstrated regulation of TGF-β signaling by VHL status. VHL reduces TGF-β stability, resulting in suppression of its expression in the 786-0 ccRCC cell line [146]. In agreement with these findings, cells devoid of active *VHL* secrete more TGF-β1 than cells with functional *VHL* [149].

Pro-cancerous TGF-β effects were confirmed by antibody-mediated neutralization of TGF-β1, which led to tumor regression and inhibition of angiogenesis in a xenograft athymic mouse model [146]. Several studies aimed to investigate the mechanisms of TGF-β actions in RCC. TGF-β1 secretion was observed in a panel of ccRCC-derived cell lines (SKRC-7, SHRC-10, SKRC-17, and SKRC-52) while the functionality of the TGF-β1 pathway was confirmed through an observation of increased SMAD reporter activity in ccRCC cells treated with TGF-β1. It was also found that TGFBR1 inhibition using SB431542 compound stimulated the invasiveness of ccRCC cells. Microarray analysis revealed a signature of 157 genes regulated by TGF-β1 and correlating with poor prognosis for ccRCC patients. Many of these genes were shared by both the VHL/HIF and TGF-β1/SMAD signaling pathways. This overlap between the two pathways was further confirmed by VHL reconstitution in ccRCC cells, which attenuated both TGF-β1 secretion, as well as their responsiveness to TGF-β1-induced stimulation [150]. The links between VHL and TGF-β signaling were further confirmed be a study that analyzed angiogenic factors in formalin-fixed, paraffin-embedded ccRCC tissue samples. There was a significant correlation between expressions of VEGF, TGF-β1, and the clinical stage and nuclear grade of ccRCC tumors. Furthermore, TGF-β1 protein expression negatively correlated with microvessel density (MVD). Since high MVD values correlated with longer survival for patients with ccRCC, it may indicate that TGF-β1 overexpression leads to decreased MVD, which, in turn, might affects patients’ outcomes [151].

The role of TGF-β in the regulation of adhesion, EMT, migration, and invasion of ccRCC cells is well documented. Our previous studies showed that TGF-β1 stimulated the expression of genes involved in adhesion and extracellular matrix remodeling, including *TGFBI, COL1A1*, *COL5A1*, *COL8A1*, *FN1*, *ITGA5*, *ITGAM*, and *TIMP1* [110]. TGF-β1 stimulation of RCC cell lines leads to decrease of E-cadherin and increase of vimentin and N-cadherin, synonymous with EMT activation, which is consistent with reports in other cancers [152,153,154]. TGF-β1 also induced the expression of αν integrins and mediators of the signaling pathway, including ILK (Integrin-linked kinase) and PINCH-1, known to contribute to EMT through binding and stimulation of latent TGF-β1 [155]. This in turn results in the activation of the Src/FAK complex, and EMT progression via inhibition of cadherin-dependent cell–cell interactions. Treatment of RCC cells with the synthetic integrin αVβ3 ligand RGD stimulated enhanced E-cadherin depletion, indicating synergistic cooperation between TGF-β1 and integrins. The same study revealed that these mutual TGF-β1/RGD actions are regulated by Snail1 transcription factor. The silencing of FAK and PINCH1 inhibited cooperation between TGF-β1 and RGD [156]. TGF-β1-induced migration and invasion of ccRCC cells is mediated by fascin1 [157]. Similar results were provided for bladder cancer cells [158], indicating that this could be a universal mechanism of TGF-β1-induced tumor progression.

TGF-β contributes to the formation of RCC metastasis to bone, occurring in about 40% of renal cell carcinoma patients [159]. TGFBR1 and TGFBR2 are expressed in human RCC bone metastases (RBM) while TGF-β1 is expressed and secreted by all cell lines derived from RBM tissues. Inhibition of TGF-β1 signaling attenuates tumor growth and osteolysis in mice with ccRCC xenografts. Unexpectedly, treatment of RBM cells with TGF-β1 does not influence or even reduces proliferation [145]. This observation is consistent with previous results obtained in primary RCC [109,111,112,118] and suggests that TGF-β1 stimulation of RBM growth might be indirect, e.g., via the paracrine interplay between tumor cells and the bone microenvironment [145]. In accordance with this suggestion, analysis of RBM tissue samples revealed TGF-β-1-induced overexpression of MMP13 (Matrix metallopeptidase 13), a protein involved in degradation of the extracellular matrix (ECM) [160].

### 4.2. Penile Cancer

The molecular basis of penile cancer is poorly understood, mainly due to limited studies of these rare tumors. However, recently published molecular characterization of several cell lines derived from penile squamous cell carcinoma revealed frequent disturbances of the TGF-β signaling pathway [161]. These alterations included multiple copy number losses or gains (e.g., of *TGFB1*, *TGFB3*, *TGFBR2*, *BMPR1B*, *ACVR2B*, *SMAD4*, *SMAD2*, *SMAD1*, *SMAD7*, *PITX2*) as well as mutations of *TGFRB2* (R522X, S320X, S320X). The functional significance of these alterations, as well as their influence on penile cancer progression, await future analyses.

### 4.3. Testicular Cancer

TGF-β signaling is crucial for both the physiology and pathology of testis, contributing to its development and functioning [162,163]. Knock-out of genes encoding TGF-β1 and TGF-β2 in the mouse decreases the number of testis germ cells and seminiferous cords, respectively [164]. The development of testis and germline cells is regulated by activins and inhibins [165]. Analysis of mouse models revealed that expressions of nodal, activin, and TGF-β are regulated in a spatial and temporal manner during testis development, triggering mitotic arrest and expression of male fate markers [166]. In fetal testis, TGF-β attenuates proliferation [167], indicating that aberrances of the TGF-β pathway may lead to tumor development. Indeed, α-inhibin acts as a tumor suppressor and its knock-out results in the development of mixed or incompletely differentiated gonadal tumors, including intratubular, focally invasive gonadal stromal tumors in testis [168].

Regarding human studies, reports are limited mainly to simple analyses of the presence of components of the TGF-β signaling pathway in testicular tumors and cell lines, without comparisons with non-tumoral control tissues [169,170,171]. However, according to one study, expression of TGF-β1 and TGF-β2, as well as TGFBR1 and TGFBR2, was increased in testicular tumors compared with peritumoral non-neoplastic testis tissues [119]. TGF-β1, together with EGF and FGF4, synergistically contribute to differentiation of seminoma cells into the mixed non-seminoma-like cell type [172]. Since seminomas naturally progress into non-seminomas, this may suggest that TGF-β stimulates the progression of testicular tumors [173]. Furthermore TGF-β-induced signaling stimulates proliferation of TCam-2 seminoma cells [170].

The role of TGF-β1 in testis cancer is confirmed by analyses of its genetic variants. In a study involving 577 tumor cases and > 700 controls, the TGFB1 Ex5-73C > T variant was positively associated with TGCT risk while Ex1-282G and 509C > T variants were linked with increased risks of seminoma and non-seminoma, respectively (Purdue at al., 2007). In the TGF-β1 protein sequence, the Ex5-73C > T (rs1800472) variant results in a substitution of threonine with isoleucine (T263I) while Ex1-282G > C (rs1800471) changes the arginine to proline (P25R). The functional consequences of these alterations are unknown, although it was suggested they could influence TGF-β expression, with Ex5-73T causing a decrease, and Ex1-282G > C resulting in an increase of TGF-β protein levels. The -509C > T (rs1800469) variant does not change the TGF-β1 amino acid sequence; however, it was linked with elevated plasma concentrations of TGF-β1 [174]. The exact mechanism by which altered TGF-β functioning could affect the development of TGCT is currently unknown.

The alterations of the TGF-β pathway found in testicular cancers are also detected in other members of the TGF-β signaling pathway. A single nucleotide (thymine) insertion in SMAD4 was found in 2 out of 20 analyzed seminoma germ cell tumors [175]. This insertion resulted in a frameshift mutation that created a premature STOP codon, resulting in a loss of SMAD4 protein. The authors suggested that this mutation could be the cause of the unresponsiveness of seminoma cells to TGF-β signaling, resulting in a loss of its antiproliferative action.

A recent study showed that activin A stimulates expression of MMP2 and MMP9 in a seminoma-derived cell line [176]. MMP2 and MMP9 metalloproteinases are well-known stimulators of tumor invasion and progression, which suggests that activin/TGF-β signaling may contribute to TGCT development from GCNIS.

### 4.4. Bladder Cancer

The associations between bladder cancer’s clinical course and genetic variants of TGF-β1 and its receptors has been confirmed by several studies. TGFB1 c.29C > T substitution (rs1800470) correlates with an increased risk of bladder cancer [177]. This SNP (Single Nucleotide Polymorphism) is located in a region encoding the hydrophobic core of the TGF-β1 signal peptide and results in a substitution of proline with leucine in the 10th position of the amino acid sequence. The c.+29C results in increased TGF-β1 secretion in vitro and in vivo compared with c.+29T [178]. Chen et al. found Int7G24A (rs334354) intronic variant of the *TGFBR1* gene frequently associates with transitional cell carcinoma (TCC) of the bladder as well as renal cell carcinoma [141]. Importantly, of the 65 analyzed cases of TCC and 86 of RCCs, both heterozygous and homozygous SNP carriers had a statistically significantly increased risk of developing tumors. The same study also reported one somatic mutation, resulting in a change of serine to phenylalanine at codon 57 of *TGFBR1* [141]. Interestingly, the Int7G24A SNP is also associated with increased risks of osteosarcoma [179], colorectal, and breast cancer [180,181], suggestive of its general involvement in cancer predisposition.

The role of TGF-β signaling in BC development and progression has been extensively studied, bringing conflicting results. It is generally accepted that TGF-β1 expression is increased in bladder cancer compared with normal bladder epithelium and correlates with tumor progression, although a few studies reported the opposite (Table 1).

Highly inconsistent data were provided regarding the expressions of TGF-β receptors in bladder cancer. TGFBR1 expression was increased in invasive TCC compared with low-grade and superficial tumors, as well as tumors associated with *Schistosoma* infection [128]. However, it was also shown that a loss of *TGFBR1* expression correlates with poor prognoses of bladder cancer patients [182] while loss of *TGFBR1* and *TGFBR2* correlated with increased bladder tumor grades [183], which agrees with the decreased *TGFBR2* expression in invasive tumors compared with superficial transitional cell carcinomas [184]. The expression of TGFBR3 was generally reduced in bladder cancer tumors [185]; however, in muscle-invasive bladder cancer, TGFBR3 protein expression was increased while in non-muscle-invasive tumors, it was decreased when compared with corresponding paracarcinoma tissues.

The functional analyses of TGF-β1’s role in the development and progression of bladder cancer also brought inconsistent results. For instance, according to one report, TGF-β1 treatment of T24 bladder cancer cells inhibited proliferation and viability while inducing apoptosis, which was linked with activation of the p38 MAPK-JNK-Caspase9/8/3 pathway [186]. In contrast, another study reported that TGF-β1 stimulated proliferation, migration, and invasion of the T24 cell line, in a mechanism mediated by fascin1, a protein involved in the regulation of cell motility [158]. It is doubtful if the different TGF-β1 concentrations used in both studies could influence these opposite results ([158] used higher TGF-β1 concentrations (10 ng/mL) than those utilized by [186] (0.1–5 ng/mL)). Moreover, the latter study observed a clear dose-dependent inhibitory TGF-β1 effect [186].

TGF-β1 was reported as a powerful inhibitor of the growth of rat bladder cancer cells, with a prominent exception of several more tumorigenic and invasive cell lines that exerted TGF-β1 insensitivity [187,188]. This is in agreement with an early study in which TGF-β abolished mitogenic effects of aFGF (acidic fibroblast growth factor) in rat bladder cancer cells in vitro. Specifically, TGF-β1 inhibited aFGF-induced DNA synthesis [189]. Further analyses revealed that the loss of TGF-β1 responsiveness was linked with TGFBR1 deficiency. Restoration of TGFBR1 expression rescued TGF-β1’s ability to inhibit cell growth in vitro and attenuated the growth of bladder cancer tumors in a murine xenograft model [190,191]. In accordance with these findings, in a model of transgenic mice with COX-2 (cyclooxygenase-2)-induced transitional hyperplasia of the bladder progressing to invasive carcinoma, expressions of Tgfbi, Tgfb2, and Tgfb3 were significantly decreased compared with wild-type mice [192]. This is also in agreement with studies in bladder cancer patients. There, a loss of TGF-β1 receptors, TGFBR1 and TGFBR2, correlated with the invasive tumor stage, high grade, and lymphovascular invasion while enhanced TGF-β1 levels in bladder cancer tissues and plasma of patients were associated with tumor invasiveness [121,122].

On the other hand, multiple studies indicated that TGF-β1 and its receptors stimulate the progression of bladder cancer cells. Loss of the IQGAP1 tumor suppressor leads to increased expression of TGFBR2 and activated the TGF-β1 signaling pathway, thereby stimulating growth of human bladder cancer cells [193]. TGF-β1-stimulated migration and invasion of bladder cancer cells arise mediated by Src and FAK kinase [194] as well as transgelin (TAGLN), an actin-binding protein that stimulates colony formation, migration, and invasion, as well as epithelial–mesenchymal transition [195]. TGF-β1 can also stimulate bladder cancer progression by inducing mTORC2 signaling. Specifically, TGF-β1 activates SMAD2, which triggers mTORC2 activation, resulting in AKT phosphorylation and stimulation of the motility of bladder cancer cells [196]. TGF-β1 also stimulates bladder cancer progression by triggering Shh (sonic hedgehog signaling molecule)pathway activation. Blockade of Shh activity inhibits TGF-β1-induced migration, invasion, and clonogenic growth of bladder cancer cells. Pretreatment of bladder cancer cell lines with TGF-β1 accelerated growth of BC xenografts in mice [197].

One of the key processes linked to metastatic progression is epithelial–mesenchymal transition (EMT), in which cells lose their epithelial features and acquire a mesenchymal phenotype, detach from the basement membrane, and start to migrate and invade local and distant tissues. There is a general agreement that TGF-β induces EMT in T24 bladder carcinoma cells [198,199,200,201]. These effects are mediated by TGFBR1, as silencing of this receptor attenuates the migration and invasiveness of T24 cells, with concomitant downregulation of pro-invasive MMP9, as well as integrins α2, α3, and β1 [198]. TGF-β1-induced sumoylation of TGFBR1 is a prerequisite for induction of the epithelial–mesenchymal transition in bladder cancer cells [202]. This process is reversed by SENP2 (SUMO-specific protease-2), which removes SUMO from TGFBR1, thereby inhibiting TGF-β1-induced EMT, resulting in inhibition of the invasion of bladder cancer cells in vitro and tumor metastasis in vivo. The expression of SENP2 in bladder cancer patients is reduced, leading to attenuation of its suppressive effects on tumor progression. As a result, bladder cancer patients with reduced SENP2 expression have a greater chance for the development of more aggressive tumors and poor outcomes [202]. TGF-β1 actions in bladder cancer are also mediated by ARMc8 (Armadillo repeat-containing protein 8). Specifically, ARMc8 mediates TGF-β1-induced migration and invasion, as well as epithelial–mesenchymal transition [203]. Pro-cancerous TGF-β effects are counteracted by protein phosphatase PPM1A, which dephosphorylates SMAD2/3 TGF-β effectors. Loss of PPM1A promotes TGF-β1-induced EMT in vitro and correlates with bladder cancer progression and poor prognosis for patients [199]. TGF-β1 signaling is crucial in the context of the tumor microenvironment. EMT, migration, and invasion of bladder cancer cells are induced by TGF-β1 secreted by cancer-associated fibroblasts (CAFs), with ZEB2NAT, long non-coding RNA mediating TGF-β1-induced protumorous effects [201]. TGF-β1-induced EMT can be also mediated by other lncRNAs, such as Malat1 [204]. Silencing of Malat1 attenuates TGF-β1-induced migration and invasion of bladder cancer cells and inhibits progression of tumor xenografts in mice [204].

Interestingly, it was suggested that TGF-β1 could promote EMT in bladder cancer progression by altering the expression of genes involved in the synthesis of glycans that mediate cell-to-cell adhesion [205]. Specifically, treatment of HCV29 cells with TGF-β1 reduced the expression of α-mannosidase 2 and α-L-fucosidase, resulting in decreased levels of bi-, tri-, and tetra-antennary complex N-glycans; increased expression of hybrid-type N-glycans; and increased levels of fucosylated N-glycans [205].

The involvement of TGF-β1-signaling in the pathogenesis of bladder cancer is also confirmed by altered expression of its downstream effectors. Accordingly, the expression of TGFBI (TGF-β1-induced) is enhanced in muscle-invasive bladder cancers (MIBCs) compared to non-muscle invasive tumors (NMBCs) and correlates with poor survival of patients. TGFBI is secreted by bladder cancer cells to induce proliferation, migration, and invasion, as well as EMT in an autocrine manner. Moreover, TGFBI overexpression stimulates the growth of bladder cancer xenografts in mice [206]. Interestingly, enhanced TGF-β1 expression was suggested as a mechanism mediating cancerous effects of chronic long-term low-dose ionizing radiation exposure [207]. TGF-β1 expression was elevated in BC patients of the Chernobyl region compared with patients not exposed to radiation. The authors suggested that TGF-β1 could act as a sensor of excess ROS production resulting from radiation exposure [207].

TGF-β1 also acts as a mediator of other EMT-inducing proteins. For instance, EIF5A2 stimulates TGF-β1 expression via STAT3 to induce EMT and stimulate migration and invasion [208] while cancer-associated fibroblasts (CAFs) secrete Kindlin-2, which induces EMT in a TGF-β-dependent manner [209]. The TGF-β1/SMAD2 pathway is also utilized by Golgi membrane protein 73 (GP73) to induce EMT and promote invasion and metastasis of bladder cancer [210]. TGF-β1 mediates signaling initiated by collagens. Huang et al. showed that expression of COL6A3 is increased in bladder tumors, stimulates proliferation and angiopoiesis as well as epithelial–mesenchymal transition, with possible involvement of TGF-β. COL6A3 silencing resulted in reduced expression of TGF-β as well as phosphorylation of SMAD2 and SMAD3 [211]. Trim59, the expression of which is upregulated in bladder tumors, promotes proliferation, EMT, migration, and invasion of bladder cancer cells. Silencing of Trim59 attenuates migration and invasion of bladder cancer cells while the presence of TGF-β1 relieves the suppressive effect of Trim59 knock-out [212].

There is strong evidence that TGF-β signaling contributes to the tumor microenvironment and affects anti-tumor immunity. As revealed by in vivo studies in rabbits, the growth and progression of bladder cancer is promoted by mesenchymal stem/stromal cells (MSCs) that stimulate secretion of growth factors and ECM-remodeling proteins, including TGF-β1 [213]. The tumor-growth stimulatory effects of MSC are mediated by TGF-β receptor and SMAD2 protein [214]. This particular study, however, has several limitations, including the lack of a direct evaluation of TGF-β receptor and SMAD2 expression following silencing [214]. It is thus difficult to conclude to what extent the silencing of both genes contributed to the attenuation of MSC tumor growth stimulatory effects. Intensified tumor-derived TGF-β secretion facilitates evasion of immune surveillance. Specifically, in patients with superficial transitional cell carcinoma, increased plasma TGF-β1 attenuates cytotoxicity of NK (Natural Killer) cells [215]. High expressions of TGF-β1 and TGFBR2 attenuate T cell penetration into the center of metastatic urothelial carcinoma tumors [216], bringing the rationale for combination therapies involving immune checkpoint inhibitors and TGF-β1 blockers (further discussed in Chapter 6). Furthermore, it was found that TGF-β mediates S1P1-induced tumor-associated expansion of Treg cells in bladder cancer patients. S1P1 is a receptor of sphingosine-1-phosphate, a lipid involved in the regulation of the tumor immune microenvironment. Specifically, S1P1 triggers phosphorylation of SMAD2/3, which activates the TGF-β signaling pathway and production of TGF-β by bladder cancer cells. This in turn results in enhanced production of Tregs from naive T cells [217]. Interestingly, most (up to 86%) of TGF-β1 secreted by bladder cancer cells is encapsulated in exosomes that target healthy fibroblasts and stimulate their differentiation into cancer-associated fibroblasts (CAFs) by triggering SMAD2 phosphorylation [218]. Interestingly, the expression of TGF-β1 and TGFBR1 in peripheral blood mononuclear cells (PBMNCs) of bladder cancer patients is increased compared with healthy individuals, which coincides with the role of TGF-β1 signaling in bladder cancer immunity. According to the same study, however, the levels of TGFBR1 in urine sediments of bladder cancer patients were decreased, with a concomitant increase of TGF-β1, which indicates independent mechanisms of TGF-β1 signaling in cancer and immune cells [219].

The importance of TGF-β receptors in bladder cancer is underscored by studies that demonstrated that Tgfbr2 knock-out or Tgfbr1 inhibition attenuates growth and progression of chemically-induced bladder tumors in mice [220] while TGFBR3 knock-down in a human T24 bladder cancer cell line results in reduced viability, colony formation, migration, and invasion [185]. Deletion of Tgfbr2 decreased the population of cancer stem cells, attenuated proliferation, and induced apoptosis of bladder cancer cells in vivo. Furthermore, conditional knock-out of Tgfbr2 decreased EMT as indicated by lowered expression of mesenchymal markers, including vimentin, Slug, Snai1, Twist, and Zeb1, with concomitant upregulation of epithelial E-cadherin [220]. Notably, TGFBR2 mutations are frequently found in bladder cancer patients. Glu269 to Lys mutation (G→A) facilitates TGF-β1-induced invasion of bladder cancer cells [221].

The expressions of other members of the TGF-β protein family associate with the tumorigenicity of cell lines derived from bladder cancer xenografts in mice [222]. Accordingly, the expression of BMP2 was increased in a tumorigenic and invasive cell line compared with nontumorigenic cells while inhibin-βB expression was enhanced in invasive bladder cancer cell lines compared with nontumorigenic and tumorigenic cells. The promoter of *BAMBI* (BMP and activin membrane-bound inhibitor) is hypermethylated in a subset of high-grade bladder cancers, leading to its low expression [222,223]. BAMBI acts as a TGFR1/BMPR1-related pseudoreceptor and interferes with TGF-β/BMP signaling by hindering the formation of functional receptor complexes. In accordance with this function, forced BAMBI expression attenuated the migration of cancer cells induced by TGF-β1 or BMP2 [223].

BMP9 is overexpressed in bladder cancer and stimulates proliferation and migration of cancer cells by increasing the expression of lncRNA UCA1, which in turn leads to activation of AKT. BMP9 also stimulates the growth of bladder cancer tumors in vivo [224]. Another member of bone morphogenetic proteins (BMPs), the growth differentiation factor-9 (GDF-9), was suggested as a possible tumor suppressor in bladder cancer. Specifically, ectopic expression of GDF-9 in bladder cancer cell lines attenuated cell growth, and reduced migration and adhesion. The study also suggested that GDF-9 expression, while clearly present in normal bladder tissue, was absent from bladder cancer tissue samples [225]. A similar tumor suppressive function was also found for GDF-15, which inhibited the proliferation and invasion of bladder cancer cells in vitro, and attenuated the growth of bladder cancer xenografts in vivo. Remarkably, GDF-15 knock-out resulted in reverse effects. These effects were mediated by GDF-15-induced blocking of EMT and polarization of F-actin. The GDF-15 evaluation in bladder cancer cell lines suggested that its expression could be reduced in bladder tumors, possibly due to DNA hypermethylation as well p53 inactivation in bladder cancer cells [226].

### 4.5. Prostate Cancer

The role of TGF-β in prostate tumors is a broad topic that has been extensively earlier reviewed [227,228,229,230], thus here we only provide a short outline of the mechanism of TGF-β actions in prostatic cancer cells. 

TGF-β signaling regulates proliferation, growth, differentiation, and apoptosis of prostatic stromal and epithelial cells [231,232]. As in the other genitourinary cancers, altered expression of TGF-β1 family proteins has been observed in prostate tumors. Increased TGF-β1 levels were found in tumor tissue samples [130,131,132,133,134,135], serum [136], and urine [133] of PCa patients. TGF-β1 overexpression correlates with the prostate cancer stage and grade, patients’ survival rate as well as the degree of angiogenesis induction and the presence of bone metastasis [134,233]. Downregulation or loss of TGF-β receptors was observed in about 30% of cases of prostate cancer compared with normal prostate tissues while expressions of TGFBR1 and TGFBR2 were lower in metastatic compared with primary tumors [234]. Upregulation of TGFBR2 enables pro-apoptotic activity of TGF-β1 and inhibits the growth of prostate cancer cells through a caspase-1-mediated mechanism, whereas downregulation of this receptor leads to stimulation of malignant transformation [235,236]. The resistance of prostate cancer cells to TGF-β-mediated growth inhibition is thus possibly a result of decreased expression of *TGFBR2*, which acts as a tumor suppressor gene [237,238]. Downregulation of TGFBR2 in PCa cells might result from hypoxic activation of DNA methyltransferases, which leads to the hypermethylation of the promoter region of *TGFBR2* [239]. Contrarily, earlier research reported a lack of promoter methylation and mutations in the *TGFBR2* gene [240]. TGFBR3 downregulation is the most common modification of the TGF-β signaling pathway in prostate cancer and contributes to stimulation of cancer cells’ motility and invasiveness in vitro as well as enhanced tumorigenicity in vivo [241]. In normal prostate epithelial cells, decreased TGFBR3 expression and/or activity results in morphological changes suggestive of inhibition of cell–cell contacts and stimulation of the cancer stem cell phenotype [242].

TGF-β signaling in prostate cancer interplays with the activity of the androgen receptor (AR). AR belongs to the family of nuclear receptors and is activated by testosterone and dihydrotestosterone (DHT). Following activation, cytoplasmic AR is translocated to the nucleus to regulate the expression of target genes, either directly (by binding to AREs, androgen receptor response elements in genes’ promoters) or indirectly (by interacting with transcription co-regulators). The expression of TGF-β-regulated genes in prostate adenocarcinoma cells is affected by the interaction of AR with SMAD3, which interferes with the binding of the latter to SBEs (SMAD binding elements) [243]. Furthermore, the expression of *TGFBR2* is suppressed by DHT, which attenuates the binding of Sp1 to its promoter. The resulting decreased TGFBR2 expression leads to upregulation of TGF-β-target genes, Bcl-xL and CyclinDs, caspase-3 activation, and triggering of apoptosis [244]. *AR* mutations or loss, which lead to androgen resistance in differentiated prostate cancers, contribute to TGF-β overexpression, stimulation of growth, viability, and aggressiveness of prostate cancer cells [231,245]. TGF-β and AR synergistically stimulate apoptosis in prostate cancer cells overexpressing TGFBR2. This activation is associated with AR interaction with SMAD4 proteins [246]. Treatment of PC-3 prostate cancer cells with DHT leads to inhibition of E-cadherin and β-catenin, with concomitant overexpression of N-cadherin, indicating EMT activation. These effects are possibly mediated by Snail, which is induced by DHT [247]. Moreover, TGF-β upregulates AR signaling via activation of Twist1, which results in the induction of EMT and stimulation of invasiveness of prostate cancer cells [248,249,250].

Similar to other types of tumors, TGF-β signaling induces EMT in prostate cancer. In particular, TGF-β secreted by stromal cells triggers EMT of benign prostatic hyperplasia cells by activation of SMAD signaling [251]. Tumorigenic prostate epithelial cells progressing to malignancy avoid growth-inhibiting TGF-β activity and acquire constitutive activation of the AKT pathway. In turn, AKT modulates the response of cancer cells to TGF-β by blocking nuclear translocation of SMAD3 and p21, an important mediator of TGF-β signaling, responsible for cell cycle inhibition. TGF-β-induced EMT is mediated by the PI3K/AKT pathway. Specifically, PI3K/AKT inhibition attenuates TGF-β-induced expression of vimentin, downregulation of keratin, and increased cell motility [252]. TGF-β-induced EMT is also mediated by NF-κβ. Overexpression of TGF-β leads to upregulation of vimentin and NF-κβ in prostate tumors with high and intermediate Gleason grades. Inhibition of either TGF-β or NF-κβ suppressed the invasion of cancer cells and the EMT process. These inhibitory NF-κβ effects could not be reversed by the incubation of cells with TGF-β, suggesting that these factors may synergistically induce invasion and EMT in prostate cancer cells [253]. TGF-β-mediated EMT can also be inhibited by Elf5 (through suppression of SMAD3 phosphorylation [254] or FoxA1 [255], and induced by SOX5 [105], CML, CRM1 [256], TRPM7 [257], or SENP1. Interestingly, the latter effect involves SENP1-induced deSUMOylation of SMAD4, leading to its inhibition and induction of EMT [258].

TGF-β is involved in the regulation of the prostate tumor microenvironment, formed by myofibroblasts, carcinoma-associated fibroblasts (CAFs), endothelial cells, lymphocytes, and cancer epithelial cells that promote tumor growth and progression [259]. TGF-β acts as a chemoattractant, which triggers the migration of mesenchymal stem cells (MSC) into the vicinity of prostate cancer cells insensitive to androgen. Moreover, TGF-β plays an important role in the induction of MSC trans-differentiation into CAF-like cells, which are able to induce EMT. It was therefore suggested that blocking of TGF-β signaling in the prostate tumor microenvironment could inhibit cancer progression [260]. TGF-β was also shown to recruit immunosuppressive Treg cells in the prostate tumor environment [261]. Furthermore, TGF-β induces expression of vascular endothelial growth factor (VEGF), a crucial proangiogenic agent, in a hypoxia-dependent manner [262,263].

TGF-β is a crucial stimulator of prostate cancer metastases to bone by promoting the growth and survival of metastasizing cancer cells [264]. Suppression of TGF-β signaling in prostate cancer cells results in inhibition of its metastases to bone [265,266,267]. Knock-out of *Tgfbr1* in mice inhibits bone metastases of prostate cancer cells by disrupting their interactions with the bone microenvironment. TGF-β upregulates the expression of multiple genes involved in the progression of bone metastases, including *PTHRP*, *IL11*, and *PMEPA1*. Remarkably, *PMEAP1* knock-out induces TGF-β signaling and the formation of bone metastases, indicating negative feedback regulation between the two genes [267]. The stimulatory TGF-β effects on prostate cancer progression are counteracted by PICK1. Specifically, PICK1 upregulation in prostate cancer cells inhibited nuclear translocation of pSMAD2/3 and suppressed the expression of TGF-β target genes both in the presence and absence of TGF-β. Moreover, overexpression of PICK1 reduced the motility and invasiveness of prostate cancer cells. In accordance with these findings, the expression of PICK1 decreases in prostate cancer cells metastasizing to bone and negatively correlates with PSA levels, Gleason grade, and the presence of bone metastasis in patients. In vivo experiments conducted on male nude mice inoculated with PC-3 cells overexpressing PICK1 led to a reduction of metastatic foci, osteolytic areas of metastatic tumors along with prolonged overall survival of the animals and delayed bone metastasis formation [268].

## 5. MicroRNAs and TGF-β Signaling in GC

**Figure 2:**
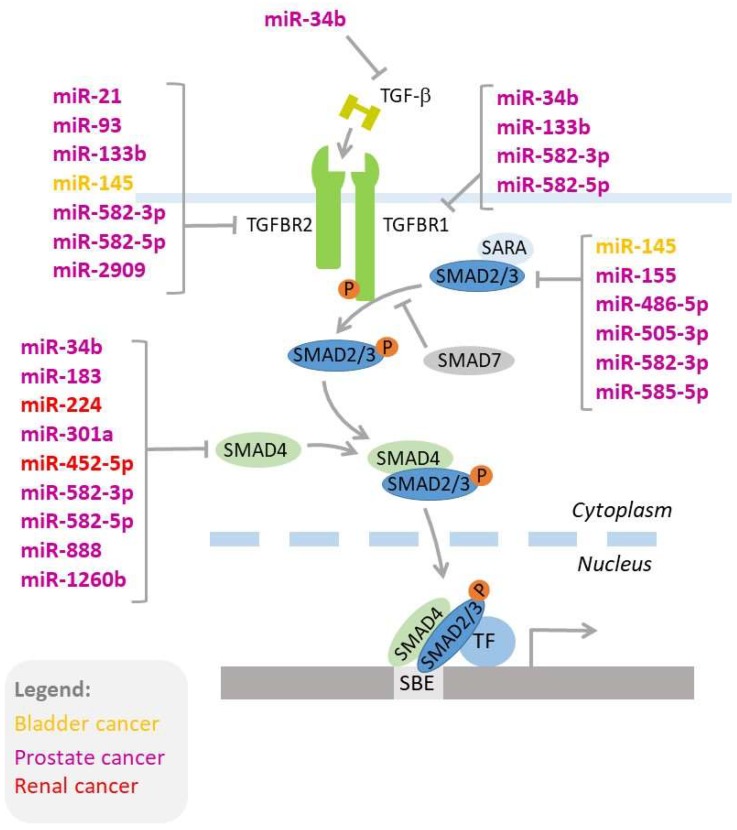
MicroRNAs regulating key genes of the TGF-β signaling pathway. MicroRNAs acting in bladder, prostate, and renal cancer are shown in colors (yellow, purple, and red, respectively). The details of miRNA-mediated regulation of the TGF-β signaling pathway are provided in the text

TGF-β signaling is regulated by miRNAs. Mechanistically, there are three types of interactions between TGF-β signaling and microRNAs: i) TGF-β regulates expressions of miRNAs, ii) miRNAs regulate the expression of genes involved in TGF-β signaling (Figure 2), and iii) miRNAs interfere with the TGF-β-induced process, such as EMT [269,270,271,272,273]. In the following chapters, we discuss the importance of these complex networks of interactions that contribute to the development and progression of genitourinary cancers, as well as responses to therapy.

### 5.1. Renal Cancer

The potential significance of miRNAs as diagnostic and prognostic biomarkers in renal cell carcinoma has been confirmed by multiple studies [274,275,276]. These small molecules participate in the regulation of RCC cell growth, cell cycle, apoptosis, angiogenesis, tissue invasion, and metastasis [277]. Noteworthy, miRNAs may be useful markers, allowing for the selection of patients to treatment. For instance, increased serum levels of miR-183 correlate with patients’ resistance to NK cytotoxicity [278] while miR-942 confers resistance of RCC patients to sunitinib [279]. It was also shown that miR-381 enhances the sensitivity of RCC cells to 5-fluorouracil [280].

Most studies addressing the interplay between TGF-β and microRNAs in renal cancer focus on miRNA-mediated regulation of the expression of genes involved in the TGF-β signaling pathway. Several reports showed that miRNAs stimulate ccRCC progression by targeting SMAD4. Accordingly, miR-19a, the expression of which is enhanced in ccRCC tumors, activates proliferation of ccRCC cells while suppressing the expression of SMAD4 and PTEN. Furthermore, the expression of miR-19a significantly correlated with the tumor stage and poor prognosis of ccRCC patients [281]. miR-452-5p stimulates migration and invasion of RCC cells by decreasing SMAD4 expression. Interestingly, these effects are counteracted by treatment with sunitinib, which attenuates migration and invasion of RCC cells by suppressing miR-452-5p expression. Increased miR-452-5p expression is associated with a poor prognosis for RCC patients [282]. SMAD4 and SMAD5 are also directly targeted by miR-224, which induces degradation of their transcripts [283]. Furthermore, miRNAs can also regulate SMAD4 expression indirectly. ccRCC tumors overexpress miR-629, which targets TRIMP33 (tripartite motif-containing 33), an inhibitor of the TGF-β/SMAD signaling pathway. TRIMP33 regulates SMAD4 expression and cellular localization or competes with SMAD4 for binding with SMAD2/3. Inhibition of miR-629 results in suppression of TGF-β-dependent induction of SMAD2/3 and SMAD4 through upregulation of TRIM33 expression. In contrast, transfection of RCC cells with miR-629 mimic potentiated the impact of TGF-β on EMT, motility, and invasion [284]. ACVR2B, another member of the TGF-β signaling pathway, is regulated by miR-192, miR-215, miR-194, miR-141, and miR-200c in nephroblastomas. The expression of these miRNAs is decreased in these pediatric tumors compared with mature kidneys [285]. TAK1 (TGF-β-activated kinase 1) is directly targeted by miR-486-5p in RCC. Remarkably, induced expression of TAK1 leads to stimulation of tumor growth, which can be inhibited by overexpression of miR-486-5p [286].

miRNAs can also interfere with TGF-β-induced signaling in renal cancer cells. Treatment of RCC cell lines with TGF-β1 induces the expression of *RBL2*, a member of the Rb family involved in TGF-β1-dependent inhibition of proliferation and cell cycle progression. RBL2 is also directly targeted by miR-93, which downregulates its expression. This in turn prevents TGF-β-induced activation of *RBL2* expression. Consequently, miR-93 abolishes tumor-suppressive TGF-β1 effects, providing an explanation for TGF-β1 resistance in RCC. Intriguingly, TGF-β1 reduces miR-93 expression, which leads to increased *RBL2* expression, indicating reciprocal feedback regulation in the TGF-β1-RBL2-miR-93 axis [287]. Furthermore, miR-429 disrupts TGF-β-dependent inhibition of E-cadherin expression, suggestive of an attenuation of TGF-β-induced EMT. miR-429 expression is decreased in ccRCC tumors and may function as a potential tumor suppressor [288].

Remarkably, TGF-β1 controls the expression of miRNAs involved in the regulation of cellular adhesion in ccRCC. Treatment of RCC cells with TGF-β1 inhibits expressions of miR-30a-5p and miR-328 while increasing miR-25-3p levels, contributing to an altered expression of adhesion proteins, including COL5A1 and ITGA5, and changes in the adhesive properties of ccRCC cells. Inhibition of miR-25-3p potentiates the TGF-β1-stimulatory effect on the expression of adhesion proteins, indicating its interference with the cellular effects of transforming growth factor [289].

### 5.2. Penile Cancer

There are limited studies that combine simultaneous TGF-β1 and microRNA analysis in penile cancers, mainly due to the rare diagnoses of these tumors. The first NGS analysis of small RNAs expressed in penile cancers was published in 2015. Interestingly, the TGF-β signaling pathway was among the top enriched pathways predicted to be targeted by miRNAs differently expressed in penile cancer tissues compared with matched adjacent non-cancerous tissues [290]. Furthermore, TGFB3 and TGFBR2 were found within the top pathways involving miRNA-targeted genes disrupted in penile cancer [291]. *TGFBR2* was also predicted as one of the 47 candidate driver genes targeted by multiple miRNAs, the expressions of which were altered in penile cancer samples compared with normal penile tissue [292]. None of these studies, however, included functional analysis to validate the miRNA-mediated regulation of TGF-β pathway genes, nor the TGF-β-mediated regulation of miRNAs. Thus, the functional links between TGF-β and miRNAs and their role in penile cancer pathogenesis await future analyses.

### 5.3. Testicular Cancer

Multiple studies revealed altered expressions of miRNA in TC, both tumor tissue expressed and circulating in plasma/serum [293]. In particular, circulating miRNAs offer great promise as potential prognostic markers and are currently explored in clinical trials focusing on TGCT [293]. Surprisingly, the studies addressing the interplay between TGF-β signaling and miRNAs in testicular cancer are scarce. The global analysis of 782 miRNAs in germ-cell tumors revealed that TGF-β signaling was one of the two predicted pathways most highly targeted by miRNAs that were differentially expressed in yolk sack tumors (YSTs) compared with germinomatous tumors (GERs). The expressions of 34 genes of the TGF-β/BMP signaling pathway (including ligands, receptors, SMAD proteins, key target genes) were altered in YST compared with GER. Based on this study, however, it is difficult to draw conclusions regarding testicular tumors because the YST group included tumors located not only in testis but also liver, peri-rectum, and ovary [171]. The functional associations between TGF-β signaling and miRNAs in testis are supported by a study showing TGF-β-induced changes in miRNAs expressions in a mouse GC-spg cell line derived from spermatogonia [294]. It can thus be expected, that disturbed TGF-β pathway functioning in testicular cancer should be associated with altered expressions of target miRNAs. This hypothesis, however, awaits future experimental evaluation.

### 5.4. Bladder Cancer

The functional associations between TGF-β signaling and microRNAs in bladder cancer were initially supported by a study showing significant correlations between expressions of miRNAs detected in urine sediments of bladder cancer patients and transcripts of genes involved in the TGF-β signaling pathway [295]. Later reports revealed that microRNAs could be both mediators and modulators of TGF-β effects in bladder cancer cells. TGF-β1 stimulates the expression of ZEB1-AS1, which acts as a sponge of tumor-suppressive miR-200b, leading to a decrease of its expression. As a result, the expression of the miR-200b target, fascin1, is induced in bladder cancer cells, leading to activated migration and invasion. Induced ZEB1-AS1 expression inhibits apoptosis, promotes the cell cycle, and activates proliferation, as well as stimulates the growth of bladder cancer xenografts in mice [296]. According to another study, miR-200b targets and inhibits the expression of MMP16. TGF-β1-mediated repression miR-200b leads to activation of MMP16 expression and stimulation of the migration of bladder cancer cells [297]. TGF-β1-induced expression of oncogenic miR-221 triggers EMT and stimulates the migration and invasion of bladder cancer cells [298]. On the other hand, microRNAs can interfere with TGF-β signaling. miR-520f, which is capable of reversing EMT in bladder cancer cells, downregulates expression of TGFBR2 [299].

The miRNA-TGF-β interference also contributes to the chemoresistance of bladder cancer cells (further discussed in Chapter 6).

### 5.5. Prostate Cancer

There are multiple studies showing altered expression of microRNAs in prostate cancer tissues and urine, suggesting their potential as diagnostic and prognostic biomarkers [300,301,302]. miRNAs are also involved in the regulation of the AR signaling pathway. For instance, miR-125b overexpression is associated with androgen-independent growth of PCa cells in castrate mice [303]. 

The interplay between TGF-β signaling and miRNA activity in prostate cancer has been documented by multiple studies. miR-15a/16 target and downregulate expressions of SMAD3 and ACVR2A, resulting in attenuated expression of TGF-β dependent genes, including MMP2, E-cadherin, Snail, and Twist, and leading to inhibition of EMT and invasion of prostate cancer cells [304]. Prostate tumors overexpress the TR4 transcription regulator, which attenuates the expression of miR-373-3p, leading to enhanced invasion of prostate cancer cells. Remarkably, miR-373-3p is capable of inhibiting the expression of TGFBR2 and its downstream pSMAD3. In consequence, TR4 knock-out facilitates miR-373-3p-mediated downregulation of TGFBR2 and pSMAD3. Inoculation of mice with prostate cancer cells overexpressing both miR-373-3p and TR4 results in the development of metastases, suggesting that silencing of miR-373-3p and/or TR4 might be used in prostate cancer treatment [305]. The expression of TGFBR2 is also regulated by miR-93. During hypoxia, miR-93 expression is induced, resulting in downregulation of TGFBR2. The expression of miR-93 is increased in prostate tumors compared with non-tumorous control samples and correlates with cancer progression. Transfection of prostate cancer cell lines with miR-93 mimics stimulates their proliferation, migration, and invasion [239]. Complex regulation of the TGF-β signaling pathway is exerted by miR-34b, which modulates expressions of TGF-β, TGFBR1, and pSMAD4, p53. Transfection of PC3 prostate cancer cells with miR-34b mimics results in the inhibition of cell growth, migration, and invasion [306]. The expression of SMAD4 is also regulated by multiple other microRNAs, including miR-1260b, miR-301a, miR-888, and miR-183 [307,308,309,310,311]. Interestingly, expression of miR-1260b is downregulated by genistein, an isoflavone naturally occurring in plants (e.g., lupin, fava beans, soybeans, and coffee), exerting chemopreventive and anticancer activities [312,313,314]. Genistein can also directly suppress SMAD4 expression by inducing changes in DNA methylation and histone modifications [307]. miR-301a represses the expression of SMAD4, promoting the proliferation of prostate cancer cells in vitro and tumor growth in vivo. Interestingly, the expression of miR-301a is regulated by high glucose levels [308], which fits the known association between hyperglycemia and the development of prostate cancer, as well as the prostate cancer grade, Gleason score, and increased risk of prostate cancer recurrence [315,316]. MicroRNAs also regulate the expression of SMAD2. Overexpression of miR-486-5p in prostate cancer results in downregulation of SMAD2 while promoting proliferation, migration, and colony formation. In vivo, inhibition of miR-486-5p attenuates tumor development [317]. Decreased expression of miR-133b in prostate tumors and bone metastases leads to upregulation of TGFBR1 and TGFBR2, enabling TGF-β signaling, which contributes to the migration and invasion of prostate cancer cells. Importantly, the induction of miR-133b expression inhibits the formation of prostate cancer bone metastasis in the mouse model [318]. Similarly, decreased expression of miR-505-3p contributes to prostate cancer progression by targeting SMAD2 and SMAD3, and stimulating migration and invasion. Furthermore, miR-505-3p expression in prostate cancer bone metastases negatively correlates with serum PSA, Gleason grade, and bone metastatic-free survival of patients [319].

TGF-β signaling can also be indirectly regulated by miRNAs. For instance, miR-539 interferes with TGF-β-induced EMT activation by targeting DLX1, a transcription factor that stimulates the expression of SMAD4. The expression of miR-539 in prostate tumors is decreased, contributing to elevated expression of DLX1, which in turn facilitates TGF-β-induced changes in expression of e-cadherin, vimentin, Snail1, and Slug, synonymous with epithelial–mesenchymal transition. Re-introduction of miR-539 into prostate cancer cells inhibits expression of DLX1, leading to attenuation of TGF-β signaling, inhibition of proliferation, migration, and invasion, as well as growth of prostate cancer xenografts in mice [320]. TGF-β signaling in prostate cancer is regulated by a complex axis involving AR, miR-2909, and STAT1. Specifically, miR-2909 attenuates the expression of SOC3, a negative regulator of STAT1, resulting in elevated expression of the latter and leading to overexpression of SMAD7. This in turn results in decreased phosphorylation and activation of SMAD3, contributing to inhibition of TGF-β signaling. On the other hand, miR-2909 directly targets and reduces the expression of TGFBR2, which disables pSMAD2/3 activation, resulting in enhanced expressions of c-Myc and CCND1, reduced p21CIP, and escape of TGF-β suppressive effects on proliferation and viability. Furthermore, miR-2909 and androgen receptor are involved in a positive feedback regulation in which AR stimulates the expression of miR-2909 while miR-2909 enhances the expression of AR, providing further reinforcement of TGF-β signaling inhibition [321]. Strikingly, similar positive feedback was found between AR and miR-21, which targets and downregulates TGFBR2, preventing TGF-β antitumor activity [322]. miR-331-3p stimulates TGF-β1-induced EMT by decreasing expressions of NRP2 and NACC1, multifunctional proteins involved in cancerous transformation and progression. Specifically, a miR-331-3p-induced decrease of NRP2 and NACC1 results in upregulations of TGF-β1 and SMAD4, triggering EMT of prostate cancer cells [323]. Furthermore, loss of tumor suppressive miR-19a-3p leads to activation of SMAD2 and SMAD4, contributing to the formation of bone metastasis in a mouse model [324]. Loss of miR-132/212 in prostate cancer results in upregulation of targeted SOX4, thereby contributing to activation of TGF-β signaling and induction of EMT. Re-expression of miR-132/212 in prostate cancer cells suppresses their invasion and migration, and attenuates colony formation by inhibiting TGF-β-induced EMT [325]. Interestingly, the crosstalk between TGF-β and microRNAs is also utilized by the tumor microenvironment for stimulation of cancer progression. Accordingly, it was shown that pre-adipocytes induce miR-301a expression in prostate cancer cells, leading to suppression of its target, AR, and subsequent activation of TGF-β/SMAD/MMP-9 signaling and promotion of cancer invasiveness in vivo [326].

The spatio-temporal changes in the expression of microRNAs may provide a mechanical explanation for the “TGF-β paradox” in prostate cancer. It was found that expressions of miR-582-3p and miR-582-5p are increased in prostate tumors compared with adjacent normal tumors while being downregulated in bone metastatic tissue compared with metastases in other organs. Remarkably, overexpression of these microRNAs repressed the migration and invasion of prostate cancer cells in vitro and the formation of bone metastases in vivo. These effects were mediated by microRNA-induced repression of key mediators of the TGF-β signaling pathway, including TGFBR1, TGFBR2, SMAD2, and SMAD4, which led to the reprogramming of multiple genes involved in the formation of bone metastases [327]. Loss of miR-15 and miR-16 in prostate cancer cells potentiates TGF-β signaling by upregulating *USP9X* (a gene encoding an enzyme deubiquitinating SMAD4), as well as activin RIIA, an activin receptor, contributing to the survival of cancer cells in bone marrow and the formation of bone metastasis. Furthermore, these effects synergize with the activity of miR-21, the enhanced expression of which results in suppression of SMAD7. As a result, depletion of miR-15/miR-16 with concomitant upregulation of miR-21 in prostate cancer cells stimulates the formation of bone lesions in mice [328]. Remarkably, TGF-β upregulates miR-21 expression by stimulating the processing of its primary transcript by ribonuclease Drosha [329]. This suggests that the reciprocal feedback regulation between miRNAs and TGF-β in prostate cancer may be involved in the regulation of metastasis.

Interestingly, TGF-β-mediated regulation of microRNAs emerges as an important mechanism contributing to the progression of prostate cancer. In this regard, activin A is a powerful regulator of miRNAs, triggering changes in the expression of nine microRNAs (miR-222-3p, miR-15b-5p, miR-93-5p, miR-18a-5p, miR-30a/30d-5p, let-7c, and miR-196b-5p). Activin A is a member of the TGF-β signaling pathway, recognized as an important negative regulator of the growth and migration of prostate cancer cells [330]. TGF-β regulates the expression of miR-96 via activation of the SMAD 2/3/4 complex, which interacts with SBEs (SMAD2/3-binding elements) within the promoter region of pri-miR-96 precursor and stimulates its expression. miR-96 regulates the expression of AKT1S1, which inhibits mTOR and promotes the proliferation and formation of bone metastasis in mice [331]. TGF-β attenuates the expression of miR-1 and miR-200b, leading to increased expression of Slug, EMT stimulation, and progression of prostate cancer in a mice model. Remarkably, this regulation involves a negative feedback circuit in which microRNAs inhibit the expression of Slug while the latter attenuates the expression of microRNAs in a TGF-β-dependent manner. TGF-β-induced reduction of miR-1 and miR-200b disrupts this miRNA-Slug balance and triggers a cascade of changes in the expression of genes involved in EMT and cancer progression [332]. Interestingly, it was suggested that the miR-200 family and miR-205 could oppose the reversal of EMT in a benign prostatic hyperplasia epithelial cell line [333].

## 6. TGF-β1 and microRNAs and Treatment of GC

The TGF-β signaling pathway is apparently an attractive option for therapeutic approaches in cancer. One of the early studies reporting TGF-β as a target for a therapeutic approach in urinary bladder cancer was published in 1998. In that study, antisense oligonucleotides attenuated TGF-β secretion by bladder cancer cells, reduced colony growth in soft agar, and inhibited the growth of tumors inoculated in mice. Unfortunately, that study did not include a statistical analysis of the presented data, so the conclusions remain elusive [334]. TGF-β is also a target of hispolon (6 -(3,4-dihydroxyphenyl)-4-hydroxyhexa-3,5-dien-2-one), HPL), a compound isolated from *Phellinus linteus*, exerting anticancer properties against multiple tumor types, including cancers of the cervix, colon, and kidney [335,336], as well as melanoma [337]. Hong et al. showed that HPL attenuates TGF-β-induced EMT of bladder cancer cells, resulting in reduced migration and invasion [338].

The significance of TGF-β signaling as a clinical target emerged through recent studies on the combined therapies involving targeting TGF-β and immune checkpoints. An example of such an approach is M7824, a bi-functional fusion protein consisting of avemulab, a monoclonal antibody directed against PD-L1 (programmed death-ligand 1) and the extracellular domain of TGFBR2 [339]. Avelumab acts as an immune checkpoint inhibitor, enabling the cytotoxic activity of T-cells towards tumor cells, and was approved by the FDA (Food and Drug Administration) for treatment of urothelial carcinoma in 2017 [340]. The extracellular TGFBR2 domain counteracts the functioning of all three TGF-β isoforms by acting as a ‘trap’, efficiently eliminating the TGF-β pool available for endogenous TGF-β receptors and thus preventing the immunosuppressive activity of high TGF-β levels produced by tumor cells. The study demonstrated that the TGFBR2 component of M7824 increased the sensitivity of urothelial transitional cell carcinoma cells towards TRAIL-mediated lysis. Compared to the sole PD-L1 blockade, M7824 also stimulated antigen-specific CD8+-mediated tumor cell lysis. Mechanistically, treatment of bladder cancer cells with M7824 altered the expression of genes involved in the angiogenesis process, remodeling of the extracellular matrix, EMT, as well as extracellular markers involved in immunogenic modulation [339].

TGF-β signaling determines patients’ responses to immunotherapies. TGF-β +C28.>T polymorphism (in other studies described as c.-1347C > T, -509C > T, and rs1800469) was linked with patients’ outcome to BCG (Bacillus Calmette–Guérin) immunotherapy. Specifically, homozygotic TT patients had a lower risk of recurrence following BCG treatment [341]. Interestingly, this genotype is associated with increased TGF-β expression when compared with CC homozygotes, and individuals with T substitution have twice higher TGF-β1 plasma concentrations when compared with CC carriers [174,178]. TGF-β modulates the functioning of myeloid-derived suppressor cells (MDSCs) of bladder cancer patients. MDSCs regulate the antitumor immune responses and suppress T-cell function. Yuan et al. found that compared with healthy controls, bladder cancer patients have an increased population of CD14+HLA-DR−/low cells, which secreted TGF-β to suppress T-cell proliferation and production of IFN-γ (interferon-gamma) [342].

The expression of TGF-β1 and TGFBR2 is increased in metastatic urothelial cancer patients who are not responding to atezolizumab, a blocker of PD-L1 [216]. In a mouse EMT6 mammary carcinoma model, therapeutic concomitant blockade of both PD-L1 and TGF-β increased tumor infiltration by T cells (in particular, CD8+ Teff cells) and significantly inhibited tumor growth. Remarkably, these effects were not observed when either PD-L1 or TGF-β inhibitors were introduced. The immunomodulatory TGF-β actions are initiated in the tumor microenvironment, not directly in tumor cells. TGF-β acts through peritumoral stromal fibroblasts to reprogram anti-tumor immunity, as indicated by altered downstream TGF-β signaling (including pSMAD2/3) in fibroblasts [216]. These findings open new possibilities for increasing the efficiency of therapies involving immune checkpoint inhibitors.

TGF-β/miRNA crosstalk is involved in the responses of cancer cells to therapies. For instance, treatment of bladder cancer cells with celecoxib, a selective inhibitor of COX-2, attenuates proliferation, migration, invasion, as well as epithelial–mesenchymal transition. These effects are mediated by increased expression of miR-145, which directly targets and downregulates expressions of TGFBR2 and SMAD3 [343]. On the other hand, miR-145 is involved in TGF-β1-induced gemcitabine resistance of bladder cancer cells. Prolonged treatment of bladder cancer cells with gemcitabine results in induced expression of TGF-β1, which triggers SMAD-mediated repression of lncRNA-LET expression. This in turn relieves lncRNA-mediated repression of NF90, a protein involved in the regulation of transcript stability. Upregulated NF90 suppresses the biogenesis of miR-145, a suppressor of cancer stemness. As a consequence, prolonged treatment of bladder cancer cells leads to enrichment of cancer stem cells via TGF-β1 activation and the resulting dysregulation of the lncRNA-LET/NF90/miR-145 axis. The potential therapeutic significance of this mechanism has been confirmed by treatment of chemoresistant tumors with a TGF-β1 inhibitor, which re-sensitized bladder cancer cells to gemcitabine [344].

MicroRNA-mediated regulation of SMAD2 is involved in the mechanism triggered by arsenic trioxide (As_2_O_3_), a potential inhibitor of prostate cancer angiogenesis, which increases miR-155 expression by inducing DNA demethylation. In turn, re-activated miR-155 inhibits TGF-β signaling by directly targeting and decreasing expression of SMAD2, which results in suppression of VEGF secretion and inhibition of angiogenesis [345].

MicroRNAs that directly target genes of the TGF-β signaling pathway are also considered as therapeutic options in the treatment of GCs. As mentioned, re-introduction of both miR-582-3p and miR-582-5p in prostate cancer cells attenuated migration and invasion in vitro, and reduced the formation of bone metastases in vivo, by targeting SMAD2, SMAD4, TGFBRI, and TGFBRII, and leading to an inhibition of TGF-β signaling. In prostate cancer patients, decreased expressions of miR-582-3p and miR-582-5p correlated with poor bone metastasis-free survival, indicating that these miRNAs could be considered as a therapeutic option for prostate cancer patients [327].

## 7. Conclusions

Both the TGF-β signaling pathway and microRNAs clearly contribute to the development and progression of genitourinary cancers. In particular, the interplay between microRNAs and TGF-β signaling may provide a mechanistic explanation for the TGF-β paradox universally occurring in cancer cells. In the first model, microRNAs targeting key mediators of the TGF-β signaling pathway, including TGF-β receptors and SMADs, suppress pro-cancerous TGF-β actions in primary tumors. During cancer progression, the expression of these miRNAs becomes downregulated, releasing the expression of downstream TGF-β effectors and promoting metastatic growth. According to the second model, TGF-β actions may be mediated by miRNAs that either promote or inhibit cancerous progression by direct targeting of genes regulating cancerous proliferation, adhesion, migration, and invasion. In primary tumors, higher expression of tumor-suppressive miRNA overcomes the effects of oncogenic miRNAs, attenuating tumor growth. In metastatic tumors, increased expression of oncogenic miRNAs overcomes the effects of suppressive miRNAs, leading to cancer progression. Thus, in both models, the “switching” of miRNAs’ expression between primary and secondary tumor lesions would be the key mechanism contributing to the TGF-β paradox in cancer.

Clinically, the most relevant are findings on the role of TGF-β1 signaling in modulating the tumor microenvironment and immunity. Changes in the expression of TGF-β1 and microRNAs have potential as informative diagnostic and prognostic biomarkers. However, further studies are needed to validate the obtained data, in particular regarding the expressions of TGF-β1 and microRNAs in serum and urine. The interplay between the TGF-β signaling pathway and microRNAs emerges as an important field for therapeutic interventions. Therapeutic microRNAs are already being tested in clinical trials [346]. It can thus be expected that miRNAs targeting the TGF-β signaling pathway will be implemented in future clinical tests of new therapies for GC. Given the results of the in vivo studies, microRNAs that modulate TGF-β-mediated progression of prostate cancer offer great promise for the treatment of metastatic tumors.

## Figures and Tables

**Table 1:** Disturbances of TGF-β1 expression in genitourinary cancers.

Tumor Type	TGF-β Change (↓/↑) ^1^	Ref.
Renal Cancer	↑ in serum of RCC patients (vs. healthy donors)	[114]
	↑ in plasma of RCC patients (vs. healthy donors)	[115]
	↑ in plasma of metastatic RCC patients (vs. healthy donors)	[116]
	↑ in the peripheral blood of RCC patients (vs. healthy donors)	[117]
	↑ in tumor tissue (vs. normal tissue)	[108]
	↑ in tumor tissue (vs. normal tissue)	[118]
	↑ in tumor tissue (vs. normal tissue)	[110]
Penile Cancer	No data	No data
Testicular Cancer	↑ in tumor tissue (vs. normal tissue)	[119]
Bladder Cancer	↑ in tumor tissue (vs. normal tissue)	[120]
	↑ in tumor tissue (vs. normal tissue)	[121]
	↑ in plasma of metastatic patients (vs. healthy donors and vs. patients without metastasis)	[122]
	↑ in urine of bladder cancer patients (vs. healthy donors)	[123]
	↑ in high-grade tumor tissue (vs. low-grade tumors)	[124]
	↑ in serum of patients: i) with invasive tumors (vs. healthy donors); ii) with high-grade tumors (vs. low-grade tumors)	[125]
	↑ in tumor tissue of recurrent patients (v.s non-recurrent patients)	[126]
	↑ in tumor tissue (vs. normal tissue); ↑ in low-grade tumor tissue (vs. high grade); ↑ in superficial BC (vs. invasive BC)	[127]
	↑ in tumor tissue (vs. chronic cystitis)	[128]
	↓ in serum of BC patients (vs. healthy donors)	[129]
Prostate Cancer	↑ in tumor tissue (vs. normal tissue)	[130]
	↑ in tumor tissue (vs. normal tissue)	[131]
	↑ in tumor tissue (vs. normal tissue)	[132]
	↑ in tumor tissue (vs. normal tissue)	[133]
	↑ in tumor tissue (vs. normal tissue)	[134]
	↑ in tumor tissue (vs. normal tissue)	[135]
	↑ in serum of patients with PCa lymph node and/or distant metastases	[136]
	↑ in urine of PCa patients	[133]

^1^ ↑ increased expression/↓ decreased expression.
